# JS-K, a nitric oxide donor, induces autophagy as a complementary mechanism inhibiting ovarian cancer

**DOI:** 10.1186/s12885-019-5619-z

**Published:** 2019-07-01

**Authors:** Bin Liu, Xiaojie Huang, Yifang Li, Weiguo Liao, Mingyi Li, Yi Liu, Rongrong He, Du Feng, Runzhi Zhu, Hiroshi Kurihara

**Affiliations:** 1 College of Pharmacy, Jinan University, Guangzhou, 510632 Guangdong China; 2 Laboratory of Hepatobiliary Surgery, The Affiliated Hospital of Guangdong Medical University, Zhanjiang, 524001 Guangdong China; 3 School of Pharmacy, Shenyang Pharmaceutical University, Shenyang, 110016 Liaoning China; 4 Key Laboratory of Protein Modification and Degradation, School of Basic Medical Sciences, Affiliated Cancer Hospital & Institute, Guangzhou, Medical University, Guangzhou, 511436 Guangdong China; 5 Center for Cell Therapy, The Affiliated Hospital of Jiangsu University, Zhenjiang, 212001 Jiangsu China

**Keywords:** Ovarian cancer, JS-K, Reactive oxygen species (ROS), Apoptosis, Autophagy

## Abstract

**Background:**

Ovarian cancer (OC) is the second most frequent gynecological cancer and is associated with a poor prognosis because OC progression is often asymptoma-tic and is detected at a late stage. There remains an urgent need for novel targeted therapies to improve clinical outcomes in ovarian cancer. As a nitric oxide prodrug, JS-K is reported highly cytotoxic to human cancer cells such as acute myeloid leukemia, multiple myeloma and breast cancer. This study is aim to investigate the influence of JS-K on proliferation and apoptosis in ovarian cancer cells and explored possible autophagy-related mechanisms, which will contribute to future ovarian cancer therapy and supply theory support that JS-K holds great promise as a novel therapeutic agent against ovarian cancer.

**Methods:**

The cytotoxicity, extracellular ROS/RNS activity and apoptotic effect of JS-K and indicated inhibitors on ovarian cancer cells in vitro were evaluated by MTT assay, extracellular ROS/RNS assay, caspases activities assay and western blot. Further autophagy effect of JS-K and indicated inhibitors were examined by MTT assay, cell transfection, immunofluorescence analysis, transmission electron microscopy (TEM) analysis and western blot on ovarian cancer cells in vitro. In vivo, the BALB/c-nude female mice with SKOV3 ovarian cancer cells xenograft were used to examine the efficacy of JS-K treatment on tumor growth. PCNA and p62 proteins were analyzed by immunohistochemistry.

**Results:**

In vitro, JS-K inhibited the proliferation of ovarian cancer cells, induced apoptosis and cell nucleus shrinkage, enhanced the enzymatic activity of caspase-3/7/8/9, and significantly increased the production of ROS/RNS in ovarian cancer A2780 and SKOV3 cells, these effects were attenuated by inhibition of NAC. In addition, JS-K induced autophagy-related proteins and autophagosomes changes in ovarian cancer A2780 and SKOV3 cells. In vivo, JS-K inhibited tumor growth, decreased p62 protein expression and increased the expression levels of PCNA in xenograft models which were established using SKOV3 ovarian cancer cells.

**Conclusion:**

Taken together, we demonstrated that ROS/RNS stress-mediated apoptosis and autophagy are mechanisms by which SKOV3 cells undergo cell death after treatment with JS-K in vitro. Moreover, JS-K inhibited SKOV3 tumor growth in vivo. An alternative therapeutic approach for triggering cell death in cancer cells could constitute a useful multimodal therapies for treating ovarian cancer, which is known for its resistance to apoptosis-inducing drugs.

## Background

Ovarian cancer is the most common cause of gynecologic cancer-related deaths worldwide. In 2019, 22,530 new ovarian cancer cases and 13,980 ovarian cancer deaths are projected to occur in United States [1]. Moreover, in 2015, there were 521,000 new cases of ovarian cancer, and 225,000 women died of this disease in China [2]. Chemotherapy is a common method used to treat advanced ovarian cancer. However, the complications and severe side effects caused by current anticancer drugs (such as hematological and gastrointestinal toxicities) have been major problems in clinical treatments. Therefore, there is an urgent need for novel drug therapies that are effective and less toxic.

NO donor drugs have been reported to induce apoptosis in several types of human cancer cells [3]. NO prodrugs, such as O_2_-(2, 4-dinitrophenyl) 1- [(4-ethoxycarbonyl) piperazin-1-yl] diazen-1-ium-1, 2-diolate (JS-K, C_13_H_16_N_6_O_8_; CAS-No.: 205432–12-8; Fig. 1a), are a growing class of promising NO-based therapeutics. Previous studies have reported that JS-K exerts anti-tumor activities in many cancers, such as human leukemia, hepatoma, renal cancer cell lines, bladder cancer, and prostate cancer [4–8]. In vitro*,* experiments in various tumors cells involved the mitogen-activated protein kinase pathway, a regulatory mechanism, which modulated cell death, motility and proliferation [9]. The cGMP, a secondary messenger, is a vital mediator of NO for the physiological effects that NO activates soluble guanylyl cyclase to increase the production of cGMP [10]. JS-K exerts anti-tumor activities via ROS-triggered stress in non-small cell lung cancer cells and malignant gliomas [11, 12].

**Fig. 1:**
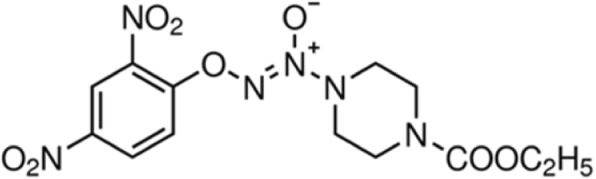
The chemical structure of JS-K

Reactive oxygen species (ROS) and reactive nitrogen species (RNS) participate in some important physiological processes such as cell survival and cell death. ROS/RNS in high levels mainly induce cell death, low levels of ROS/RNS directly regulate the activities of p53, nuclear factor-κB (NF-κB), transcription factors, nuclear factor (erythroid-derived 2)-like 2 (Nrf2), and huge protein kinase cascades that are involved in modulating the cross-talk between apoptosis and autophagy [13]. Apoptosis and autophagy are two evolutionarily conserved processes that maintain homeostasis during stress. Although the two pathways utilize fundamentally distinct machinery, apoptosis and autophagy are highly interconnected and share many key regulators [14].

Autophagy is a mechanism by which cellular material is delivered to lysosomes for degradation allowing basal turnover of cell components and providing energy and macromolecular precursors. Autophagy, a tumor suppression mechanism, has been involved in various anticancer treatments used in clinical today and many therapies that are during the research [15]. Therefore, it is significant to manipulate autophagy for the development of cancer treatment. Autophagy is usually monitored by measuring the levels of autophagy-related proteins, such as microtubule-associated protein 1 light chain 3 II (LC3II) [16]. Sequestosome 1 (p62/SQSTM1), which is a vital selective receptor for autophagy, is definitely degraded in the process of autophagy [17, 18]. Therefore, the research on the combination of p62 levels and LC3II formations can suitably reflect autophagy levels.

It was reported that JS-K induced autophagy in breast cancer cells. Electron microscopy confirmed that JS-K-treated breast cancer cells underwent autophagic cell death [19]. However, whether JS-K exerts anticancer effects via autophagy for ovarian cancer is unknown clearly. Therefore, the main objective of this study was to investigate the molecular mechanisms of cell death induced by NO released from the diazeniumdiolate NO donor JS-K in ovarian cancer cell lines and xenograft models. Nowadays, apoptosis is recognized as the major mechanism underpinning the effectiveness of anticancer therapies, but JS-K might provide an improved targeted therapeutic strategy for cancer chemotherapy.

## Methods

### Materials

JS-K was purchased from Sigma-Aldrich (St. Louis, Mo, USA). Stock solutions of JS-K (20 mM) were prepared in dimethyl sulfoxide (DMSO) and stored at − 20 °C. The stock solutions were further diluted in relative culture medium prior to the experiments. Reactive oxygen species (ROS) inhibitor NAC (N-acetyl-L-cysteine) was purchased from Abcam (Cambridge, MA, USA). Autophagy inhibitors Bafilymycin A1 (BAF) and 3-Methyladenine (3-MA) was purchased from Selleck Chemicals (USA). Cisplatin was bought from Hansoh Pharma (China). The anti-LC3B and anti-ATG-5 antibody were obtained from Sigma-Aldrich (St. Louis, MO, USA), anti-p62 antibody was bought from Abcam (Cambridge, UK), and other antibodies were purchased from Cell Signaling Technology (Danvers, MA, USA).

### Cell culture

HO8910, HO8910-PM, A2780, and SKOV3 cell lines were provided by the Affiliated Hospital of Guangdong Medical University (Zhanjiang, China). A2780 was cultured in RPMI 1640 media (Gibco, China) containing 10% fetal bovine serum (FBS, Capricorn Scientific, Germany) and 1% penicillin/streptomycin (HyClone Laboratories, USA; 100 units/mL penicillin and 100 μg/mL treptomycin). HO8910, HO8910-PM, and SKOV3 cells were cultured in McCoy’s 5A medium (Boster Biological Technology Co., Wuhan, China) supplemented with 10% FBS, 100 units/mL of penicillin, and 100 μg/mL of streptomycin. Cells were grown in a 37 °C incubator (Thermo, USA) with a humidified mixture of 5% CO_2_: 95% air. During the logarithmic phase, the cells were used for experiments.

### Cell viability

To evaluate the growth inhibitory effect of the indicated JS-K treatments, a colorimetric MTT assay was performed as described previously. Briefly, cells were seeded in 96-well plates in 0.1 mL of their respective media. Cells were plated at different cell densities because the cells had different sizes and growth rates. HO8910, HO8910-PM, and A2780 cells were plated at an initial density of 7000 cells per well. SKOV3 cells were seeded at a density of 8000 cells per well. Cells were incubated in 96-well plates overnight and then cultured with the indicated treatments (JS-K + X groups were subjected to co-treatment with 2.5 μM JS-K and indicated X) for 24 or 48 h. Thereafter, 20 μL of MTT (thiazolyl blue tetrazolium bromide, 5 mg/mL) was added during the last 4 h of incubation. The supernatants were removed, and 100 μL of DMSO was added to dissolve the formazan. Absorbance values were measured at 492 nm using a Multiskan MK3 microplate reader (Thermo Electron Corporation, USA). Cell viability was determined as a percentage of proliferating cells in the treated versus cells (Control, 100%).

### Apoptosis assay

For the detection of apoptotic cells, which performed by the Annexin V, FITC Apoptosis Detection Kit (Dojindo Laboratories, Japan), in accordance with the manufacturer’s instructions. Cells were seeded at 3.5 × 10^5^ cells/well for the SKOV3 cell line and 4 × 10^5^ cells/well for the A2780 cell line using 6-well plates. The cells were then treated with JS-K or NAC at appropriate concentrations as described (1.25–5 μM JS-K; vehicle, 2.5 μM JS-K, 200 μM NAC, co-treatment with 2.5 μM JS-K and 200 μM NAC). The cells were washed twice with PBS, and then 1× Binding Buffer was added to achieve the concentration of 1 × 10^6^ cells/mL. Next, 100 μL of each cells solution were transferred to a flow cytometric tube, and 5 μL of FITC Annexin V and 5 μL of propidium iodide (PI) were added to each tube. Each sample was gently mixed, protected from light, and stained for 15 min at RT. Finally, 400 μL of 1× Binding Buffer was added to each tube to stop the staining reaction, followed by flow cytometric analysis using the BD FACSCantoTMIIFlow Cytometer (BD Biosciences, USA). Data analysis was performed using the system’s software (BD Biosciences, USA). The percentage of cells positive for PI and/or Annexin V-FITC was reported inside the quadrants.

### Caspase enzymatic activity assay

The quantification of caspase enzymatic activity is regarded as an important readout for apoptosis. We used the Caspase-Glo 3/7 Assay Kit, the Caspase-Glo 8 Assay Kit, and the Caspase-Glo 9 Assay Kit (Promega Co., USA) to examine the activity of caspase-3/7, caspase-8, and caspase-9, respectively, according to the manufacturer’s protocol. Briefly, A2780 and SKOV3 cells were plated at a density of 4 × 10^5^ and 3.5 × 10^5^ cells per well in 6-well plates, respectively. Following the indicated treatments (1.25–5 μM JS-K; vehicle, 2.5 μM JS-K, 200 μM NAC, co-treatment with 2.5 μM JS-K and 200 μM NAC) and incubation period (48 h), approximately 6 × 10^4^ cells per sample were transferred into 1.5-mL tubes. Next, 100 μL of Gaspase-Glo reagent (relative Caspase substrate) was added to each sample, and the cells were incubated for 1 h at RT protected from light. Sample luminescence values were measured using the Sirius L Tube Luminometer (Berthold Detection Systems, Germany). Relative caspase activities were normalized to the raw luminescence units (RLUs) of the untreated control.

### Colony suppression assay

The colony-forming ability of cells was assessed as described. In these experiments, cells were plated in 6-well plates and incubated with JS-K (0, 1.25 and 2.5 μM) or the indicated treatments (vehicle, 2.5 μM JS-K, 200 μM NAC, co-treatment with 2.5 μM JS-K and 200 μM NAC) for 6 h. The cells were then harvested and quantified for each group. Around 2000 SKOV3 cells and 5000 A2780 cells per group were plated into individual wells of 6-well plates with a drug-free medium in order to assay colony formation. We changed the medium every 3 d. After 8 d of plating, the colonies were fixed in 4% paraformaldehyde (PFA) for 20 min, washed twice in PBS, stained with 0.5% crystal violet solution for 10 min, washed in ultrapure water three times, and photographed.

### ROS/RNS detection

The intracellular ROS/RNS levels were analyzed using the Cellular ROS/RNS Detection Assay Kit (Abcam, USA; catalog number: ab139473) according to the manufacturer’s instructions. For the fluorescence microplate assay, cells were plated in 6-well plates. After the indicated treatment, an equal quantity of cells was washed with PBS, centrifuged at 1000 rpm for 3 min, and incubated with 1:2500 of the Oxidative Stress Detection Reagent (Green). The cells were then transferred to a 96-well plate. After 60 min of incubation at 37 °C, the fluorescence was measured using the Mithras LB 940 Multimode Microplate Reader (Berthold Technologies, Germany) at an excitation wavelength of 488 nm and an emission wavelength of 520 nm. ROS/RNS values were normalized to the fluorescence of the untreated control. For the confocal microscopy assay, cells were seeded into 35-mm dishes with 14-mm bottom wells and subjected to the indicated treatments for 24 h. Thereafter, the cells were cultured in PBS containing 1: 2500 dilution of the Oxidative Stress Detection Reagent (Green) and 1: 2500 dilution of the Superoxide Detection Reagent (Orange) at 37 °C for 60 min in the dark. The cells were then washed two times with PBS. The LEICA TCS SP5 laser confocal fluorescence microscope (Leica, Germany) was used to measure the ROS/RNS-dependent fluorescence intensity at the excitation wavelength of 490 nm and the emission wavelength of 525 nm. The superoxide-dependent fluorescence intensity was also measured at the excitation wavelength of 550 nm and the emission wavelength of 620 nm.

### Western blot analysis

The cells were seeded into 6-well culture plates at a density of 4 × 10^5^ cells/well (for the A2780 cells) or 3.5 × 10^5^ cells/well (for the SKOV3 cells). The indicated compounds were added at the specified doses, and the samples were incubated for the specified times, washed, harvested, and lysed using RIPA lysis buffer (Beyotime Biotech Inc., Nantong, China) containing with 1 mM PMSF and 30 nM okadaic acid. Equal amounts of protein (30 μg) from each sample were separated by SDS-PAGE and transferred to polyvinylidene fluoride (PVDF, Merck Millipore, Darmstadt, Germany) membranes. The blots were blocked for 1 h in 5% fat-free milk with 0.1% Tween-20 in 0.02 M TBS buffer (TBST). The blots were incubated with 5% BSA containing the appropriate primary antibodies at a 1:1000 dilution overnight at 4 °C. After incubation with the primary antibody, the membranes were washed three times with TBST and incubated with 5% fat-free milk containing with the appropriate horseradish peroxidase-conjugated secondary antibody (Sino Biological Inc., Beijing, China; dilution 1:2000) for 4–6 h at 4 °C. Immunoblots were developed using the Immobilon TM Western Chemiluminescent HRP Substrate (Millipore Corporation, Billerica, MA, USA), and they were then exposed to Kodak film (Kodak, Rochester, NY, USA). Equal protein loading was confirmed by probing blots with the anti-GAPDH antibody.

### Cell transfection

The cells were grown on 35-mm glass-bottomed dishes (In Vitro Scientific). Subsequently, 1 μg/well of the GFP-LC3 plasmid was transfected into the cells using Lipofectamine™ 2000 (Life Technologies, USA) in Opti-MEM (Life Technologies, USA) according to the manufacturer’s instructions. After 5 h, the medium was exchanged for the standard culture medium, and the cells were incubated overnight. Then cells were treated with or without JS-K (2.5 μM) for 24 h and fixed with 4% PFA. The location and expression of LC3-GFP were observed using the LEICA TCS SP5IIconfocal microscope (Leica, Germany). For siRNA transfection, SKOV3 cells were seeded onto 6-well plates and grown to 80% confluence. Next, 0.1 μM of *ATG5*-specific siRNAs was transiently transfected into the cells using Lipofectamine™ 2000 according to the manufacturer’s recommendations. SiRNA Oligos were chemically synthesized by GenePharma (Suzhou, China; 1^#^ *ATG5*-specific siRNA sense strand: 5′-gacguugguaacugacaaatt-3′; 2^#^ *ATG5*-specific siRNA sense strand: 5′-guccaucuaaggaugcaautt-3′; 3^#^ *ATG5*-specific siRNA sense strand: 5′-gaccuuucauucagaagcutt-3′; negative control sense strand, 5′-uucuccgaacgugucacgutt-3′). After 48 h of culture, cells were treated with or without JS-K (2.5 μM) for 24 h, photographed, and harvested for Western blot analysis.

### Transmission electron microscopy (TEM) analysis

After treatment with the vehicle or 2.5 μM JS-K for 24 h, the cells were harvested using 0.25% trypsin and centrifuged at 800 rpm for 2 min. The supernatant was removed, and the cells were fixed in 3% glutaraldehyde at 4 °C for 4 h followed by post-fixing in 1% OsO_4_ for 1.5 h at 4 °C. Following ethanol dehydration and resin embedding, ultra-thin sections (90 nm) were prepared using a UC7 microtome (Leica, Germany), and the sections were then mounted on copper grids. The autophagosomes were observed using the JEM-1400 electron microscope (JEOL, Japan), and images were collected using 832 Digital Micrograph Software (Gatan, USA).

### Xenograft model and treatments

Five-week-old BALB/c-nude female mice (Lingchang biotechnology co., Shanghai, China, No. 2013001826242) were housed with five mice per cage and bred in an SPF-level animal house with a 12-h light/12-h dark cycle, a constant temperature of 20–26 °C, and a relative humidity of 40–70%. All mice were free-fed with food and water. SKOV3 cells were prepared at a concentration of 5 × 10^6^ cells/mL. Each volume of 5 × 10^5^ SKOV3 cells was resuspended in 100 μL of medium and injected subcutaneously into the left flanks of mice. After 12 d, mice with tumor growths were randomly divided into three groups (10 mice/group) and given treatments (injected subcutaneously) of a physiological saline solution, JS-K (6 mg/kg), or Cisplatin (2 mg/kg) once every 2 d for 22 d. The body weights were recorded on the indicated days. The length and width of tumors at the indicated time points were measured using a Vernier caliper, and the volumes of the tumors were calculated using the following formula:

$$ \mathrm{Volume}\kern0.5em =\kern0.5em \left(\mathrm{width}\kern0.5em \times \kern0.5em \mathrm{length}\right)/2 $$

On the 22th day after the initiation of the drug treatments, the mice were sacrificed by treating with 5% isoflurane (Linuo Pharma, Jinan,China) in an isoflurane anesthesia system rodent anesthesia machine (Yuyan Instruments, Shanghai; ABS; 1 L/min O_2_), the xenografts were harvested, and the extraneous tissues were carefully removed. Tumors were photographed, fixed in 10% buffered formalin, and processed for paraffin embedding and sectioning. Serum aspartate aminotransferase (AST) and alanine aminotransferase (ALT) levels were quantified using an ELISA kit according to the manufacturer’s instructions.

### Histological analysis and immunohistochemistry

After fixation and routine dehydration, all tumor samples were embedded in paraffin and cut into 2-μm thick sections. The xenografted specimens for histological analysis were stained with hematoxylin and eosin (HE) in order to observe the general tissue morphology under the DM4000B microscope (Leica, Germany). For immunohistochemistry, sections were deparaffinized using xylenes for 10 min each and hydrated using a graded alcohol series (100 to 75%) for 5 min each. Antigen retrieval was performed by heating the sections in citrate buffer for 2 min in a pressure cooker. The endogenous peroxidase activity was inactivated using 0.3% hydrogen peroxide for 10 min at RT in the dark. Afterwards, sections were incubated with a 1:8000 dilution of anti-PCNA antibody and 1:800 dilution of anti-P62 antibody (Cell Signaling Technology, USA) overnight in a moist chamber at 4 °C. The next day, the sections were washed and incubated with an HRP-conjugated rabbit secondary antibody (Cell Signaling Technology, USA) for 30 min at RT. The PCNA and p62 signals were detected by using DAB substrate (brown). All tumor sections were counterstained with haematoxylin for 1 min, dehydrated, dried, and mounted using Permount TM Mounting Medium. The images were captured using the LEICA DM4000 B LED microscope (Leica, Germany).

### Statistical analysis

GraphPad Prism 5 statistical software (GraphPad Software, San Diego, CA, USA) and Microsoft Excel were used for the data analysis. Statistical analysis were performed with one-way ANOVA followed by Tukey’s test. The data were expressed as the Mean ± SEM. *p < 0.05* was considered statistically significant. Each experiment was repeated at least three times.

### Ethical statement

All animal experiments were strictly conducted in accordance with the Guide for the Care and Use of Laboratory Animals of the National Institutes of Health. Dissections were performed under anesthesia and all efforts were made to minimize suffering.

## Results

### JS-K induces cell death in ovarian cancer A2780 and SKOV3 cells

**Fig. 2:**
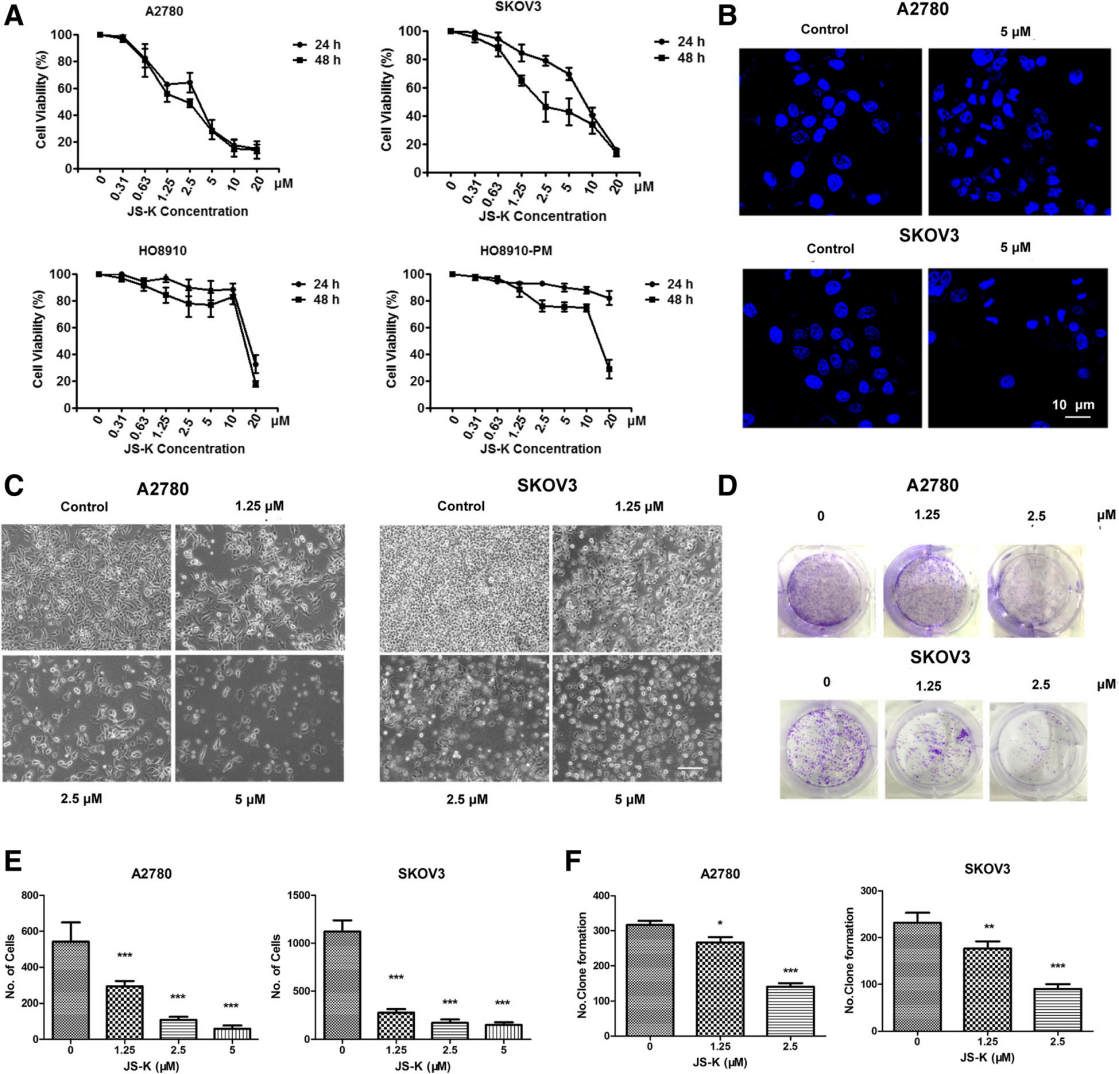
JS-K specifically induces death in ovarian cancer cells. **a** JS-K inhibition performed by JS-K in ovarian cancer cells were assessed by MTT assay. **b** Cell nucleus was stained with DAPI (48 h treatment). **c** JS-K induced cell apoptosis showing a concentration-dependent manner (200 × magnification). **d** Colony formation assay was used to detect cell proliferation of different JS-K concentration treated A2780 and SKOV3 cells. **e** and **f** The quality graphs of the **c** and **d**

**Fig. 3:**
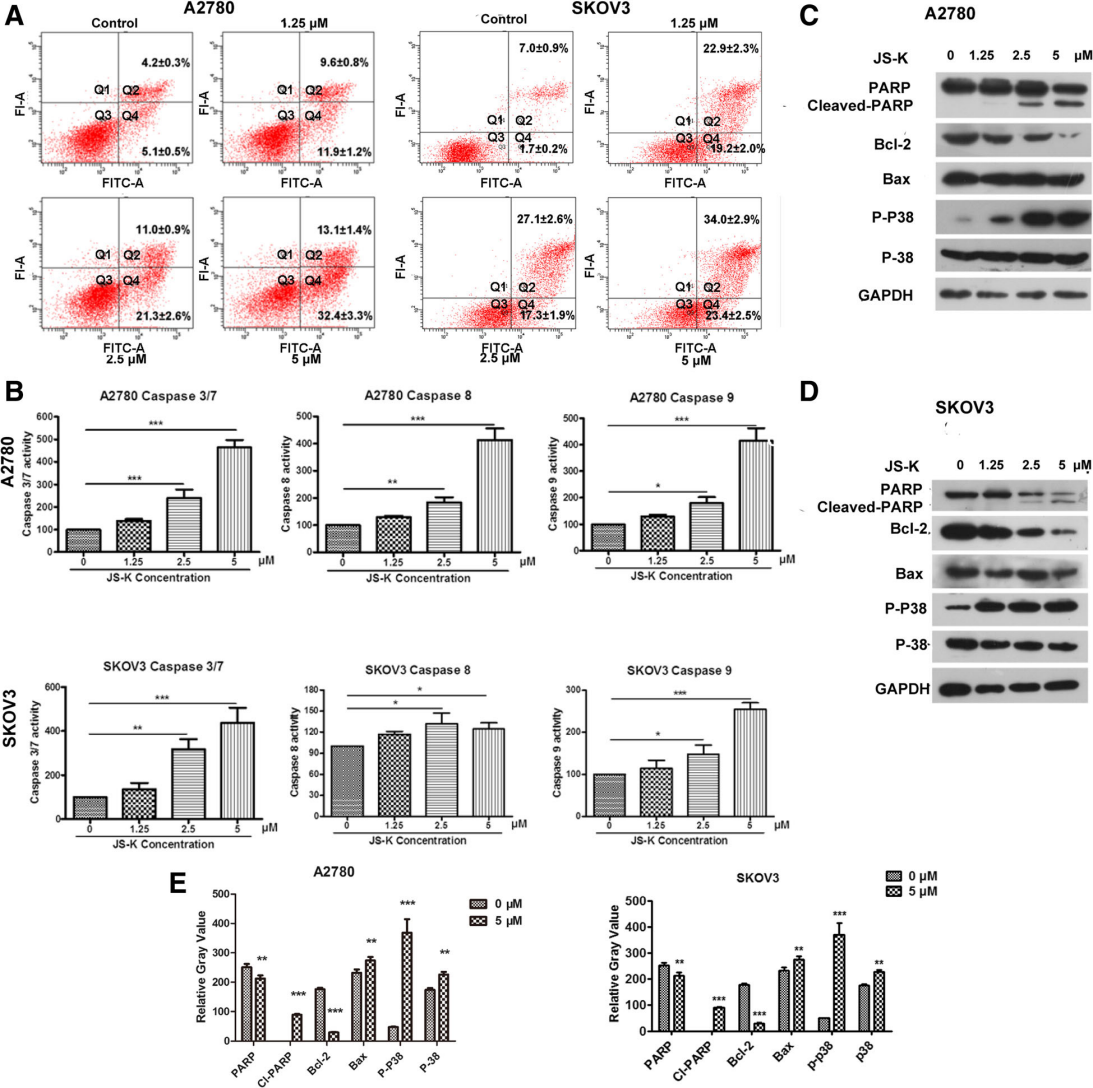
JS-K regulates survival and activates the apoptosis-related signaling pathway. **a** JS-K-induced apoptosis in A2780 and SKOV3 cells were detected by flow cytometry (Mean ± SD, 48 h and 24 h treatment). **b** Caspase family proteins 3/7/8/9 activity were detected in A2780 and SKOV3 cells treated with different concentration JS-K. **c** and **d** The expression of apoptosis-related proteins were checked in A2780 and SKOV3 cells treated with different concentration JS-K for 48 h treatment. **e** The quality graphs of the **c** and **d**

As shown in Fig. 2, JS-K inhibited the proliferation of ovarian cancer cells in a concentration- and time-dependent manner. The susceptibility to proliferation inhibition from JS-K exposure was greater in the A2780 (IC50 = 2.15 μM, 48 h) and SKOV3 (IC50 = 3.42 μM, 48 h) cell lines than the HO8910 (IC50 = 13.7 μM, 48 h) or HO8910-PM (IC50 = 15.6 μM, 48 h) cell lines, regardless of treatment durations at 24 or 48 h (Fig. 2a). Cell death was quantified using DAPI staining. The results showed that JS-K treatment induced apoptosis and cell nucleus shrinkage in the A2780 and SKOV3 cell lines (Fig. 2b). The morphology of cells treated with JS-K obviously altered, displaying a distorted shape (Fig. 2c). The colony-forming assay confirmed that the proliferation of the A2780 and SKOV3 cell lines was inhibited by JS-K in a concentration-dependent manner (Fig. 2d). In order to assess JS-K-induced apoptosis, apoptosis was detected using Annexin V fluorescein isothiocyanate (FITC)/PI. Our results revealed that JS-K increased the rates of Q2 + Q4 (early and late apoptosis rates) in a concentration-dependent manner (Fig. 3a). Furthermore, increases in the expression levels of caspase-3/7, caspase-8, and caspase-9 were observed in cells treated with JS-K, in addition to the expression levels of the apoptotic protein cleaved-PARP. In contrast, the expression levels of Bcl-2/Bax decreased in a concentration-dependent manner in JS-K-treated cells (Fig. 3b-e).

### JS-K induces cell death by producing ROS/RNS in A2780 and SKOV3 ovarian cancer cells

**Fig. 4:**
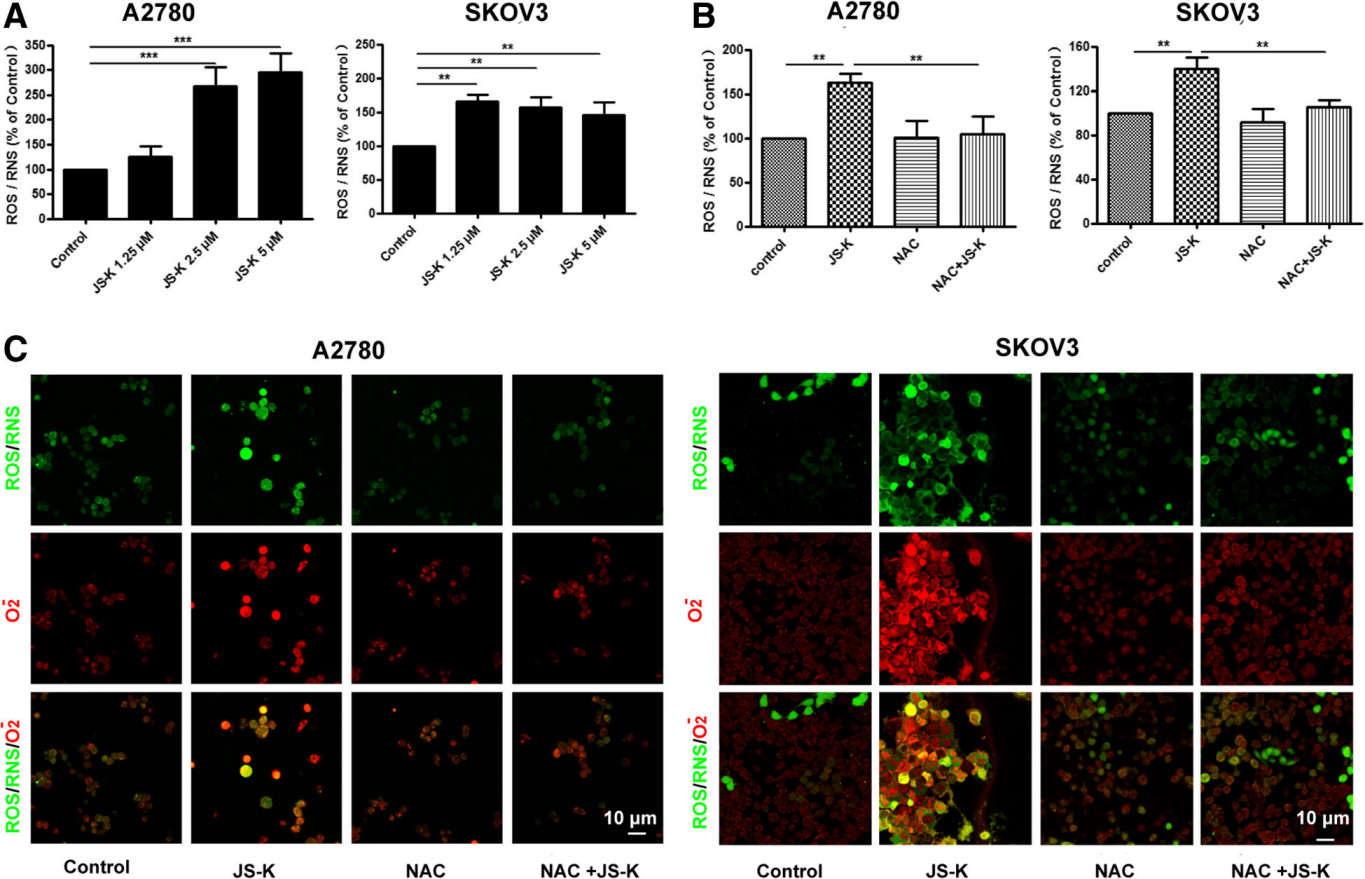
JS-K induced ROS/RNS of ovarian cancer cells could be offseted by NAC. **a** and **b** The ROS/RNS content of A2780 and SKOV3 cells treated with indicated JS-K and NAC for 48 h were analyzed by ELISA method. **b** The ROS/RNS content of A2780 and SKOV3 cells treated with indicated JS-K and NAC for 48 h were analyzed by Confocal microscopy

**Fig. 5:**
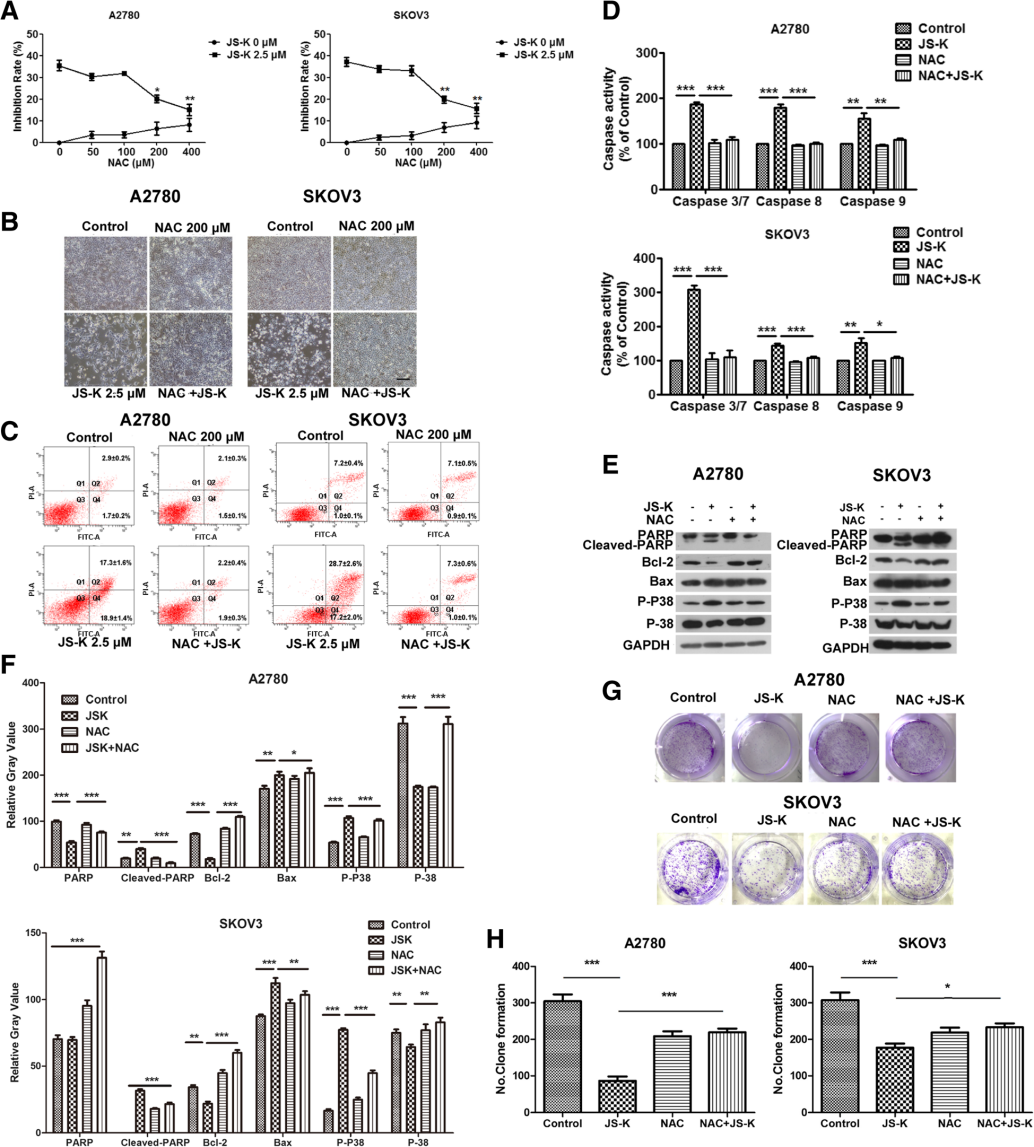
NAC rescues JS-K-mediated cell death of A2780 and SKOV3. **a** The cell viability of different NAC concentration reversed JS-K-treated A2780 and SKOV3 cells were analyzed with MTT method. **b** JS-K and NAC co-treated cells were detected with microscope (200 × magnification). **c** Annexin V/PI staining of A2780 and SKOV3 cells apoptosis treated with 2.5 μM JS-K /NAC 200 μM (Mean ± SD, 48 h and 24 h treatment). **d** The Caspase family proteins 3/7/8/9 activity in A2780 and SKOV3 cells treated with 2.5 μM JS-K or 200 μM NAC as indication. (48 h treatment) were measured using ELISA assay. **e** The expression of apoptosis-related proteins in A2780 and SKOV3 cells treated with 2.5 μM JS-K /200 μM NAC (48 h treatment) were measured using WB assay. **g** Colony formation assay was used to detect cell proliferation of JS-K and NAC co-treated A2780 and SKOV3 cells (48 h treatment). **—**represents 40 μm. (**f** and **h**) The quality graphs of the **e** and **g**

To investigate whether ROS/RNS induced by JS-K treatment could promote cell death, A2780 and SKOV3 cells were co-cultured with 2.5 μM of JS-K and different concentrations of NAC (a ROS inhibitor) for 24 h. The data demonstrated that JS-K significantly increased the production of ROS/RNS in a concentration-dependent manner, and ROS/RNS levels decreased in both cell lines co-treated with 2.5 μM of JS-K and 200 μM of NAC. This was in contrast to the increase in ROS/RNS levels observed in cells treated with 2.5 μM JS-K alone (Fig. 4a-c). A2780 and SKOV3 cells were co-cultured with 2.5 μM JS-K and different concentrations of NAC for 24 h. Cell viability was rescued by NAC at concentrations of 200 and 400 μM (Fig. 5a). Furthermore, JS-K-induced cell death was rescued by a treatment with 200 μM of NAC (Fig. 5b-c). In addition, the activity levels of caspase-3/7, caspase-8, caspase-9 and the effects of cell proliferation and colony formation in cells co-treated with JS-K and NAC were obviously reversed compared to cells treated with 2.5 μM JS-K only (Fig. 5d, g, h). Moreover, cleaved-PARP protein was not detected in cells co-treated with 2.5 μM JS-K and 200 μM NAC, while this protein was expressed in both cell lines treated with 2.5 μM JS-K alone. Meanwhile, the levels of Bcl-2/Bax increased and that of p-p38 decreased in cells co-treated with 2.5 μM JS-K and 200 μM NAC compared to cells treated with 2.5 μM JS-K only (Fig. 5e-f).

### JS-K induces autophagy in A2780 and SKOV3 ovarian cancer cells

**Fig. 6:**
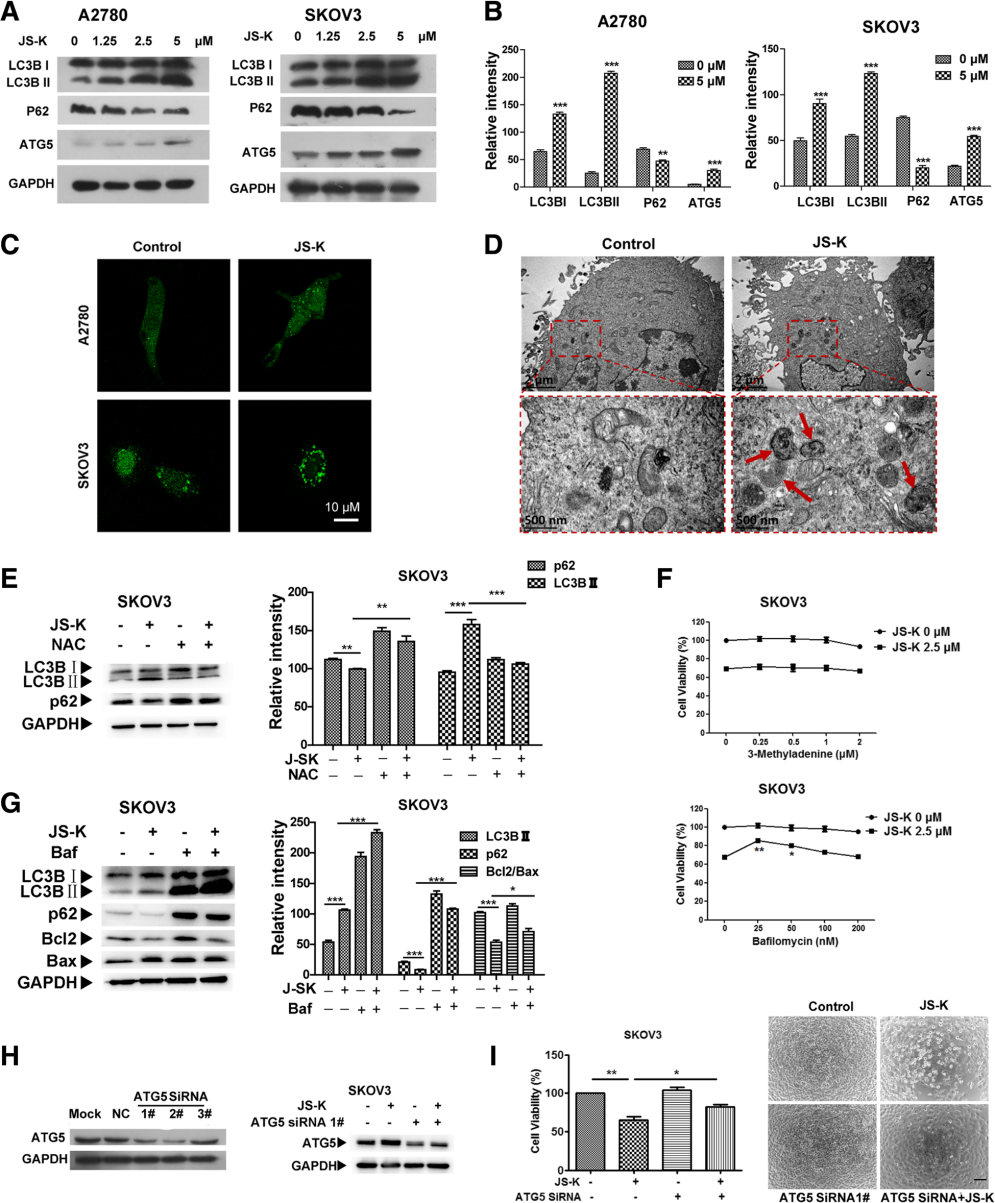
JS-K induced autophagy-related protein and autophagosome changes in ovarian cancer A2780 and SKOV3 cells. **a** Autophagy-related proteins expression were measured using WB assay. **b** The quality graphs of the A. **c** GFP - LC3 plasmid were transfected to the two cell lines, confocal microscopy was used to observe the distribution. **d** TEM was used to observe intracellular morphology (24 h treatment). **e** WB method was used to examine LC3B I/II and p62 proteins expression in SKOV3 cells treated with JS-K/NAC. **f** MTT method analyzed the cell viability of SKOV3 cells co-treated with 3-MA and BAF for 48 h. **g** WB method was used to examine LC3B I/II distribution, p62, Bcl2 and Bax proteins expression in SKOV3 cells treated with JS-K/BAF. **h** WB method analyzed ATG5 SiRNA1^#^ decreased ATG5 protein expression. **i** Microscope observed the cell morphology and MTT method analyzed the cell viability of SKOV3 cells treated with JS-K/ATG5 SiRNA1^**#**^ for 48 h. **—**represents 40 μm

In order to verify the role of autophagy, autophagy-related factors were assayed in A2780 and SKOV3 cells. The data showed that the expression of LC3BII and ATG5 proteins increased, while p62 mRNA level (data not shown) decreased and protein level expression decreased in a concentration-dependent manner in cells treated (Fig. 6a). GFP-LC3 plasmids were transfected into the two cell lines, and confocal microscopy was used to observe the distribution of LC3BI/II. Results showed that LC3BII fluorescence pattern in JS-K-treated cells was punctuate, as opposed to diffuse in control cells (Fig. 6c). Moreover, TEM was used to observe the intracellular morphology of SKOV3 cells. JS-K was found to induce a greater accumulation of autophagosomes in SKOV3 cells, which indicated a disruption in the last step of autophagy in which autophagosomes fuse with lysosomes and the cellular content is degraded (Fig. 6d). In order to verify whether autophagy could be induced by oxidative stress, we used NAC as a ROS inhibitor and performed the western blot experiment to measure the protein expression of LC3BI/II and p62 in SKOV3 cells with indicated treatments. We found the effect of which JS-K induced LC3BII protein increase and p62 protein decrease could be reversed relatively by NAC (Fig. 6e). In addition, the inhibitors 3-MA and BAF were used to identify the type of cell death induced by JS-K treatment. The results suggested that the BAF had moderately reversed JS-K-induced cell death in the SKOV3 cell line. However, 3-MA had no effect on the SKOV3 cell line (Fig. 6f). In order to confirm that JS-K induced autophagy and detect whether autophagy inhibition affects apoptosis, we performed the western blotting experiments in the SKOV3 cells with BAF. The results showed that LC3BII and BAX proteins were found to be elevated in cells co-treated with 2.5 μM JS-K and 25 nM BAF compared to cells treated with 2.5 μM JS-K alone; P62 and Bcl2 proteins were found to be increased in cells co-treated with 2.5 μM JS-K and 25 nM BAF compared to cells treated with 2.5 μM JS-K alone (Fig. 6g). To validate the effect of ATG5 in JS-K-induced cell death, Western blotting was used to show that *ATG5* siRNA decreased ATG5 protein levels in SKOV3 cells (Fig. 6h). The MTT assay was used to analyze the viability of SKOV3 cells treated with JS-K/ATG5 siRNA1^#^ for 48 h along with microscopic observations of the cellular morphology. These experiments showed that *ATG5* siRNA 1^#^ rescued the cell death triggered by JS-K treatment (Fig. 6i).

### JS-K inhibits tumor growth in vivo

**Fig. 7:**
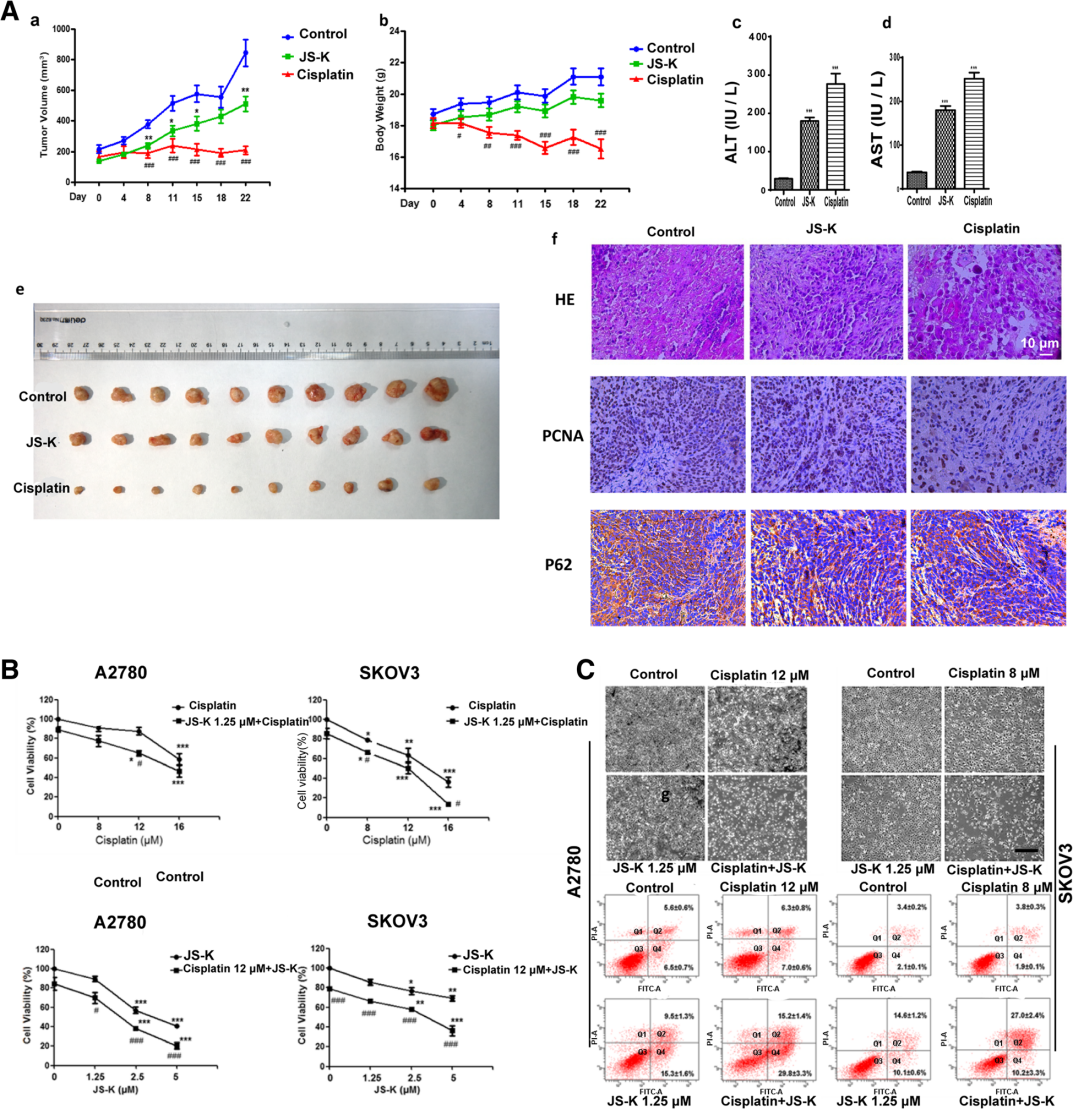
JS-K inhibits tumor growth in vivo and JS-K and Cisplatin cooperate to enhance Cisplatin sensitivity of ovarian cancer A2780 and SKOV3 cells. (**a**) *a-b*. The effect of JS-K on body weight and tumor sizes in nude mice between the Cisplatin, JS-K and control groups were compared (*n* = 10, Mean ± SD); *c-d*. AST and ALT were detected by ELISA; *e*. Cisplatin, JS-K and control groups tumor were taken picture; *f*. HE staining and Immunohistochemistry analyzed of PCNA and P62 protein expression of tumor tissue of control group, JS-K group and Cisplatin group (400 × magnification). (**b**) Cell viability was analyzed by MTT method. (**c**) Cell apoptosis were detected by microscope and flow cytometry (Mean ± SD, 48 h treatment),**—**represents 40 μm

To investigate the effects of JS-K on tumor growth in vivo, we used a mouse xenograft tumor model. Physiological saline solution (control group), JS-K (6 mg/kg), or Cisplatin (2 mg/kg) were used to treat nude mice (10 mice /group) for 23 d. The results showed that Cisplatin significantly reduced the body weights of mice and inhibited by 40% compared to the control group. JS-K treatment did not significantly impact the body weights of the mice, although the tumor volume was significantly inhibited by 75% compared to the control group (Fig. 7a-*a*, *b*, *e*). The AST and ALT levels were detected by ELISA, and JS-K was found to induce minor liver injury compared with the Cisplatin group (Fig. 7a-*c*, *d*). H&E staining demonstrated that there was a larger area of necrosis after JS-K treatment compared to the controls. Moreover, the number of cells expressing PCNA and p62 proteins in the tumors decreased in the JS-K-treated group compared to the control group (Fig. 7a-*f*). These results indicated that JS-K (6 mg/kg) inhibited tumor growth and had a fewer side effects than treatment with Cisplatin (2 mg/kg) in vivo.

### JS-K enhances cisplatin sensitivity of ovarian cancer A2780 and SKOV3 cells

We observed that Cisplatin, a classic clinical drug could induce A2780 and SKOV3 cells death. Moreover, we found that JS-K and Cisplatin cooperate to enhance Cisplatin sensitivity of ovarian cancer A2780 and SKOV3 cells (Fig. 7B-a, b). As shown in results, the effects of JS-K and Cisplatin in the A2780 and SKOV3 cell lines were investigated with MTT assay and flow cytometry, which were consistent with the microscopic observation, the rate of apoptosis of both cell lines co-treated with JS-K (2.5 μM) and Cisplatin (8 μM or 12 μM) was increased contrasted with that of cells treated with Cisplatin (8 μM or 12 μM) alone.

## Discussion

NO is known for its anti-proliferative and cytotoxic effects in glioblastoma cells, in addition to its impact on migration, invasion, and angiogenesis in various tumor cells [12, 20]. NO prodrugs, such as JS-K, release NO under enzymatic catalysis via GST isoforms [8]. Previous studies have reported that JS-K exerts anti-tumor activities in many cancers, such as human leukemia, lung cancer, colorectal cancer, bladder cancer, and prostate cancer [4, 5]. In vitro experiments indicated that some regulatory mechanisms are involved in various tumors, such as the mitogen-activated protein kinase pathways, which modulate proliferation, motility, and cell death [9]. JS-K promotes apoptosis by inducing ROS production and the ubiquitin-proteasome pathway in human prostate cancer cells [21, 22]. In this study, we showed for the first time that JS-K induced ovarian cancer cells death via an autophagic mechanism in vitro*.* It also suppressed ovarian cancer SKOV3 cells xenograft tumor growth and affected autophagy-related protein expression in vivo.

Although NO is a highly diffusible, reactive molecule and it sharply decreases in concentration in cells, we observed strong cellular effects in ovarian cancer cells after 24 and 48 h of treatment with JS-K. JS-K treatment was shown to reduce the viability of ovarian cancer cells in a concentration- and time-dependent manner. After 24 or 48 h of treatment, we observed a loss of viability in A2780 and SKOV3 cells treated with JS-K (0.31–20 μM). Thus, JS-K selectively reduced the viability of A2780 and SKOV3 cells. Apoptosis is considered to be the process of cell death induced by JS-K through activation of caspases and the fragmentation of DNA [23, 24]. Therefore, alterations in apoptosis rates and apoptosis-related proteins were investigated. As expected, we observed the activated caspase 3/7/8/9, the cleavage of PARP1 into a fragment size of 89 kDa, a reduction of Bcl2/Bax levels and an increase in P-P38 levels, all of which are normally linked to apoptosis. In this study, NAC (a common antioxidant) was used to confirm that JS-K caused ROS/RNS stress in A2780 and SKOV3 cells and determine whether JS-K-induced apoptosis could be reversed by it. The results showed that JS-K caused cell proliferation defects, caspase 3/7/8/9 activation and apoptosis-related proteins expression changes, which could be reversed by NAC. In conclusion, JS-K induced ovarian cancer A2780 and SKOV3 cells death through ROS/RNS stress-mediated apoptosis in vitro.

According to that report, excessive autophagy could induce cell apoptosis, the crosstalk between apoptosis and autophagy is complex, and autophagy can promote cell survival or cell death under various cellular conditions [14]. Both apoptosis and macroautophagy (hereafter referred to as autophagy) can be induced by extracellular stimuli, such as a treatment with a chemotherapeutic agent [25]. Autophagy-related proteins, such as LC3B and p62, were also examined. LC3BII protein increased and p62 decreased in a concentration-dependent manner, which suggested that autophagy flux may be activated by JS-K. Meanwhile, autophagosomes were observed in the cells’ ultrastructure, we found that JS-K could accelerate autophagosomes formation. The autophagy-related function of ATG5 in SKOV3 cell death induced by JS-K was confirmed. The results showed that ATG5 expression increased, and *ATG5* siRNA decreased the extent of cell death induced by JS-K. All of these results suggested that autophagy was related to SKOV3 cell death induced by JS-K, which has not been reported before. In order to verify whether autophagy could be induced by oxidative stress, we performed the western blot experiments with antioxidant NAC. As results showed, NAC inhibited the effects that JS-K induced autophagy-related proteins expression changes. That indicated that JS-K maybe caused SKOV3 cell death through ROS/RNS stress-mediated autophagy. Our findings are consistent with the apelin-13-induced MCs-HUVEC adhesion via a ROS-autophagy pathway [26]. Moreover, the autophagy inhibitors 3-MA and BAF were used to verify the autophagic effects of JS-K-induced cell death. We found that only BAF could attenuate the cell death induced by JS-K in the SKOV3 cell line. BAF is an inhibitor of the V-type ATPase as well as certain P-type ATPases. Treatment with BAF ultimately results in a block infusion of autophagosomes with lysosomes, thus preventing the maturation of autophagosomes into autolysosomes. The results of western blot also showed that BAF affected the effects JS-K caused the changes of autophagy-related proteins and Bcl2/Bax proteins. These results indicated that JS-K could induce autophagy, which might modulate the effects JS-K induced apoptosis pathway Bcl2 protein family.

Previous studies have shown that JS-K significantly reduced the growth of a variety of tumors (such as NSCLC, malignant gliomas, and multiple myeloma) compared with cells in control animals treated with vehicle [12, 27]. According to the above experimental results in vitro, we verified the anti-ovarian cancer effects of JS-K in vivo. The results showed that Cisplatin (2 mg/kg) significantly reduced the body weights of the mice, and AST and ALT levels were higher than that of the JS-K-treated group (6 mg/kg). Meanwhile, the tumor volume was inhibited by 75% compared to the control group. JS-K treatment (6 mg/kg) did not significantly impact body weights of mice, although the tumor volume was significantly inhibited by 40% compared to the control group. The morphology of the tumors, p62 and PCNA expression levels changed significantly among the three groups of tumors. PCNA protein expression in the JS-K-treated group was more than that of the Cisplatin-treated group and less than that of control group, which suggested that the tumor proliferation capacity of JS-K-treated group was poorer than that of control group, but better than that of the Cisplatin-treated group. The results showed that JS-K (6 mg/kg) has anti-tumor activity, but it has no significant impact on the growth of nude mice compared to the control group. Although Cisplatin (2 mg/kg) has an excellent tumor suppression effect, its inhibitory effects on the growth of nude mice are obvious, which is associated with a variety of clinical side effects for associated with the use of this chemotherapy. Cisplatin’s side effects include neurotoxicity, nephrotoxicity, ototoxicity, nausea, and vomiting [28]. All of the above results showed that JS-K has anti-tumor activity and maybe has fewer side effects than Cisplatin. Due to the severe side effects of Cisplatin and the drug resistance of chemotherapeutics, the search for chemotherapy-compatible drugs is a hot area of research. Accordingly, we investigated whether JS-K could potentiate the antineoplastic effect of Cisplatin, the results showed that the ovarian cancer cells SKOV3 and A2780 with co-treatment of JS-K with Cisplatin were subjected to more cell death than that with Cisplatin treatment alone. Based on our finding, JS-K may be used as compatible drugs of cisplatin to increase antitumor activity against ovarian cancer in clinical future.

## Conclusions

In this study, we demonstrated that a cell death mechanism of ovarian cancer was caused by the NO donor JS-K in vitro and in vivo. According to our research results, we have demonstrated that JS-K can induce cell death in ovarian cancer cells via ROS/RNS-mediated autophagy and ROS/RNS-triggered apoptosis pathways in vitro. In vivo, JS-K treatment has an anti-tumor effect that is less robust than that of Cisplatin treatment. The growth inhibition side effects of JS-K treatment (liver injury and weight loss) are minimal compared to that of Cisplatin treatment. In conclusion, our results suggested that JS-K suppresses tumor growth in vivo*,* and it elicits anti-tumor effects via ROS/RNS-mediated autophagy and ROS/RNS-triggered apoptosis pathways in vitro. The activation of an alternative cell death pathway could be useful for developing multimodal cancer therapies for ovarian cancer, which is known for its strong anti-apoptotic mechanisms and drug resistance.

## Abbreviations

3-MA: 3-Methyladenine
ALT: alanine aminotransferase
AST: aspartate aminotransferase
ATP: adenosine triphosphate
BAF: Bafilymycin A1
JS-K: (O2-(2,4-dinitrophenyl) 1-[(4-ethoxycarbonyl)piperazin- 1-yl]diazen-1-ium-1,2-diolate)
MAPK: mitogen-activated protein kinases
NAC: N-acetyl-L-cysteine
PARP: poly(ADP-ribose) polymerase
PCNA: proliferating cell nuclear antigen
RNS: reactive nitrogen species
ROS: reactive oxygen species
